# Acute and chronic effects of cannabinoids on effort-related decision-making and reward learning: an evaluation of the cannabis ‘amotivational’ hypotheses

**DOI:** 10.1007/s00213-016-4383-x

**Published:** 2016-09-02

**Authors:** Will Lawn, Tom P Freeman, Rebecca A Pope, Alyssa Joye, Lisa Harvey, Chandni Hindocha, Claire Mokrysz, Abigail Moss, Matthew B Wall, Michael AP Bloomfield, Ravi K Das, Celia JA Morgan, David J Nutt, H Valerie Curran

**Affiliations:** 1 Clinical Psychopharmacology Unit, University College London, Gower Street, London, WC1E 6BT UK; 2 Imanova Ltd, Burlington Danes Building, Imperial College London, Hammersmith Hospital, Du Cane Road, London, UK; 3 Division of Brain Sciences, Imperial College London, London, UK; 4 Psychiatric Imaging Group, Medical Research Council Clinical Sciences Centre, Hammersmith Hospital, London, UK; 5 Division of Psychiatry, University College London, Maple House, London, UK; 6 Psychopharmacology and Addiction Research Centre, University of Exeter, Exeter, UK; 7 Neuropsychopharmacology Unit, Division of Experimental Medicine, Imperial College London, Burlington Danes Building, Du Cane Road, London, UK

**Keywords:** Cannabis, Cannabinoids, THC, Cannabidiol, Motivation, Reward, Effort-related decision-making, Reinforcement learning, Addiction

## Abstract

**Rationale:**

Anecdotally, both acute and chronic cannabis use have been associated with apathy, amotivation, and other reward processing deficits. To date, empirical support for these effects is limited, and no previous studies have assessed both acute effects of Δ-9-tetrahydrocannabinol (THC) and cannabidiol (CBD), as well as associations with cannabis dependence.

**Objectives:**

The objectives of this study were (1) to examine acute effects of cannabis with CBD (Cann + CBD) and without CBD (Cann-CBD) on effort-related decision-making and (2) to examine associations between cannabis dependence, effort-related decision-making and reward learning.

**Methods:**

In study 1, 17 participants each received three acute vaporized treatments, namely Cann-CBD (8 mg THC), Cann + CBD (8 mg THC + 10 mg CBD) and matched placebo, followed by a 50 % dose top-up 1.5 h later, and completed the Effort Expenditure for Rewards Task (EEfRT). In study 2, 20 cannabis-dependent participants were compared with 20 non-dependent, drug-using control participants on the EEfRT and the Probabilistic Reward Task (PRT) in a non-intoxicated state.

**Results:**

Cann-CBD reduced the likelihood of high-effort choices relative to placebo (*p* = 0.042) and increased sensitivity to expected value compared to both placebo (*p* = 0.014) and Cann + CBD (*p* = 0.006). The cannabis-dependent and control groups did not differ on the EEfRT. However, the cannabis-dependent group exhibited a weaker response bias than the control group on the PRT (*p* = 0.007).

**Conclusions:**

Cannabis acutely induced a transient amotivational state and CBD influenced the effects of THC on expected value. In contrast, cannabis dependence was associated with preserved motivation alongside impaired reward learning, although confounding factors, including depression, cannot be disregarded. This is the first well powered, fully controlled study to objectively demonstrate the acute amotivational effects of THC.

**Electronic supplementary material:**

The online version of this article (doi:10.1007/s00213-016-4383-x) contains supplementary material, which is available to authorized users.

## Introduction

The endocannabinoid system, which includes the cannabinoid-1 (CB1) and cannabinoid-2 (CB2) receptors and their endogenous ligands, is putatively involved in reward processing and addiction (Curran et al. 2016; Maldonado et al. 2006; Parsons and Hurd 2015). Δ-9-Tetrahydrocannabinol (THC), the main active compound in cannabis, is a CB1 receptor partial agonist (Petitet et al. 1998), which may modestly increase dopamine release in the human striatum (Bossong et al. 2015). Dopamine is considered critical in various reward processes (Berridge and Robinson 1998; Schultz et al. 1997). Individuals who met DSM-IV criteria for cannabis dependence or abuse showed reduced striatal dopamine synthesis capacity (Bloomfield et al. 2014a), which was negatively correlated with their apathy scores (Bloomfield et al. 2014b). However, other studies have shown no difference between cannabis users and non-users in dopamine receptor density (Albrecht et al. 2013; Sevy et al. 2008; Stokes et al. 2009; Urban et al. 2012). In terms of alterations to the endocannabinoid system, cannabis dependence has been associated with reduced levels of CB1 receptors (D’Souza et al. 2015; Hirvonen et al. 2012) and reduced anandamide levels in cerebrospinal fluid (Morgan et al. 2013b).

Cannabis contains many cannabinoids, other than THC. Of particular interest is cannabidiol (CBD) which has a complex mode of action, including inhibition of the metabolism and reuptake of anandamide, inhibition of adenosine uptake, agonism of the 5-HT_1a_ receptor (McPartland et al. 2015) and agonism at the GPR55 receptor (Ryberg et al. 2007). Acute THC has dose-related amnestic (Curran et al. 2002), psychotic (Morrison et al. 2009) and anxiogenic (Morrison et al. 2009) effects. CBD has been shown to attenuate or block these negative effects (Bhattacharyya et al. 2010; Englund et al. 2013; Morgan et al. 2010b). Furthermore, CBD may have some anti-addictive properties in animals and humans ( Morgan et al. 2010a, 2013a; Ren et al. 2009), and use of high-THC/low-CBD cannabis was especially predictive of cannabis dependence, compared with other types of cannabis (Freeman and Winstock 2015). Given these opposing pharmacological and psychological effects of THC and CBD, we hypothesized that CBD may buffer the effects of THC on reward processing.

Historically, cannabis use has been associated with reduced motivation (McGlothlin and West 1968). Early, poorly controlled studies into the acute effects of cannabis found both amotivational (Miles et al. 1974) and null (Mendelson et al. 1976) effects. More recently, both pro-motivational (Foltin et al. 1990) and amotivational (Cherek et al. 2002) effects have been reported. However, the former study did not provide traditional rewards (e.g. money, food) in return for work, preferred work activities were earned instead. Furthermore, the latter study had a sample of only five participants. Hence, there is very little well-conducted, empirical research into the acute effects of cannabis on motivation to earn rewards. Moreover, to the authors’ knowledge, no one has examined the effects of CBD on motivational processing in humans.

Early studies of chronic effects of cannabis found no difference when comparing heavy with light cannabis users on fixed ratio button-pressing tasks for rewards (Mello and Mendelson 1985; Mendelson et al. 1976). Survey data has also failed to demonstrate a link between long-term cannabis use and amotivation (Barnwell et al. 2006; Musty and Kaback 1995), although cannabis use has been shown to predict anhedonia (Bovasso 2001). Daily, adolescent cannabis users had a lower motivation for monetary reward than non-users, although comorbid mental health problems and other drug use were not reported and may have confounded group differences (Lane et al. 2005). Studies that have investigated anticipatory BOLD response for monetary reward, thought to be an indicator of intact reward processing, have found opposing results, with one showing reduced (van Hell et al. 2010) and another showing enhanced (Nestor et al. 2010) striatal activation in dependent cannabis users compared to healthy controls.

Much of the research concerning the psychopharmacology of reward processing has focused on dopamine. In the past, learning about rewards (Schultz et al. 1997), vigour of responding (Niv et al. 2007), incentive-salience attribution (Flagel et al. 2011) and the pleasure taken from reward consumption (Small et al. 2003; Volkow et al. 1997) have all been linked to dopamine. Two key aspects of reward processing are effort-related decision-making (i.e. motivation) and reward learning, which have been operationalized in humans using the well-validated Effort Expenditure for Rewards Task (EEfRT) (Treadway et al. 2009) and Probabilistic Reward Task (Pizzagalli et al. 2005). Performance on both of these tasks has been investigated with regard to dopaminergic functioning (Pizzagalli et al. 2008; Wardle et al. 2011; Treadway et al. 2012b), suggesting that enhanced extracellular dopamine levels improves motivation (Wardle et al. 2011) and a reduction in phasic dopamine firing impairs reward learning (Pizzagalli et al. 2008). However, performance on neither task has been manipulated using cannabinoid drugs or correlated with cannabis dependence, despite the claims that cannabis use is associated with amotivation and reward processing impairments.

Across two experiments, we first tested the acute effects of cannabis without CBD (Cann-CBD) and with CBD (Cann + CBD) on effort-related decision-making. Second, we investigated associations between cannabis dependence, effort-related decision-making and reward learning. We hypothesized that

1. Cann-CBD would reduce motivation.
2. This effect would be weaker following Cann + CBD, i.e. CBD would buffer the amotivational effects of THC.
3. Cannabis dependence would be associated with reduced motivation and reward learning.

## Study 1

### Methods

A repeated measures, placebo-controlled, double-blind design was used to compare Cann-CBD, Cann + CBD and placebo. Participants were randomly allocated to one of three treatment order schedules, which were based on a Latin Square design. Seventeen participants^1^ (9 women) took part in the study; this sample size was adequately powered to detect drug × task interactions in a three-way crossover of d-amphetamine using the EEfRT (Wardle et al. 2011).

Inclusion criteria were as follows: aged between 18 and 70, smoked cannabis 3 times/week or less and have smoked cannabis 4 or more times in the last year. Exclusion criteria were as follows: regular negative experiences when smoking cannabis, alcohol use >5 days/week, other illicit drug use >2 times/month, current or history of psychosis and MRI contraindications.

Participants were recruited through word of mouth and all provided written informed consent. The study was approved by the University College London (UCL) ethics committee and was conducted in accordance of the Declaration of Helsinki. They were reimbursed £7.50/h and could win extra money via completion of various tasks.

### Assessments

#### Effort expenditure for rewards task (Fig. 1) (Treadway et al. 2012a)

This task tapped effort-related decision-making. Participants made a series of decisions between two different effort options: a low-effort choice, in which a small amount of money was available to be won (50p), and a high-effort choice, in which a larger amount of money was available to be won (80p, £1.00, £1.20, £1.40, £1.60, £1.80, £2.00). The low-effort choice required 30 spacebar presses with the little finger of the non-dominant hand in 7 s. The high-effort choice required 100 spacebar presses with the little finger of the non-dominant hand in 21 s. Participants were not guaranteed to win the money available if they completed the task; this was determined probabilistically. On one third of the trials there was a 12 % chance (low probability), on another third there was a 50 % chance (medium probability), and on another third there was an 88 % (high probability) chance of winning the money if they completed the required number of spacebar presses in time. The probability level applied to both the low-effort and high-effort choices.

**Fig. 1:**
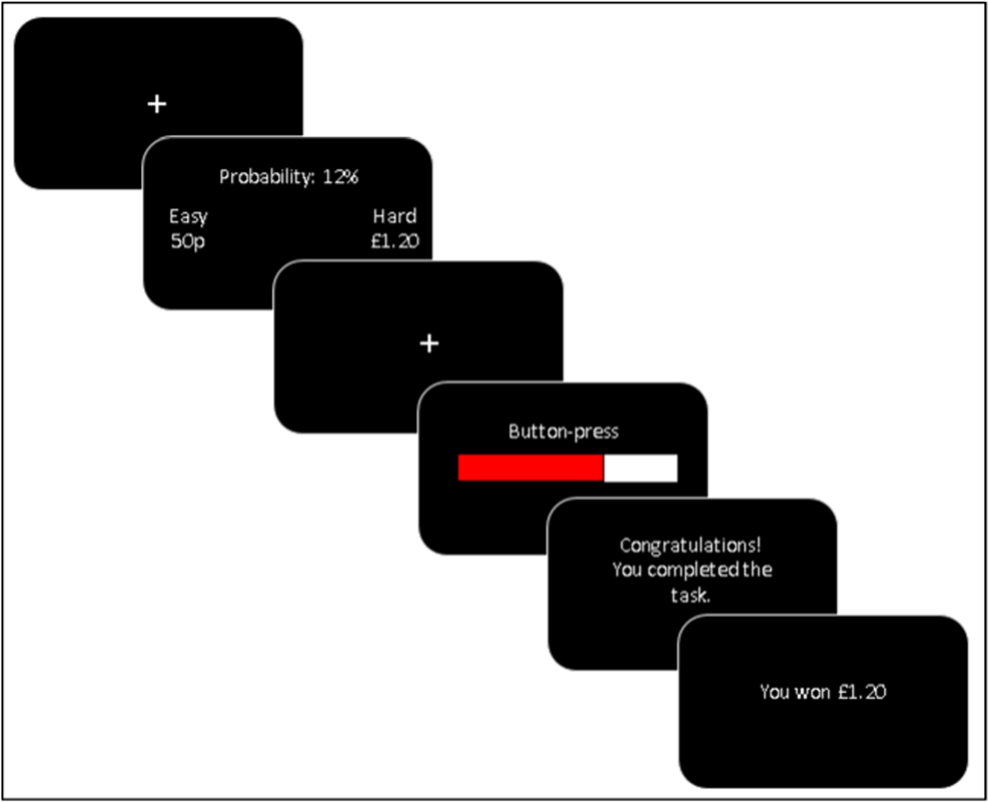
Diagrammatic representation of a single trial from the EEfRT. (1) A fixation cross is shown for 0.5 s; (2) A choice is made between a low-effort (i.e. easy) option and a high-effort (i.e. hard) option. The amount of money available to be won for both the low-effort option and the high-effort option is shown. The probability of winning the money if the subsequent button-pressing is completed is shown (this is the same for both options); (3) A fixation cross is shown for 0.5 s; (4) Button-pressing is completed for 7 s, or until 30 presses are completed (low-effort option) or 21 s, or until 100 presses are completed (high-effort option); (5) Feedback is given about whether the button-pressing was completed in time; (6) Feedback is given about whether money has been won and, if so, how much

The probability level and the amounts of money available to be won were presented on screen to the participant (see Fig. 1). Participants had 8 s to make their choice; if they did not make a choice in that time, the computer randomly selected one. Following a 0.5 s fixation cross and the spacebar-pressing stage, 2 s of feedback was given about whether the participant had successfully completed the spacebar-pressing in time, and if successful, 2 s of feedback was given about whether money had been won or not. Participants completed 21 trials in total, and the trial order was randomized. Participants kept the amounts of money won on two trials; these were randomly selected at the end of the task.

Important predictor variables in this task are probability (chance of winning on each trial if the trial is completed), magnitude (the amount of money available on the high-effort choice) and expected value (the multiplication of probability and magnitude). Furthermore, previous research has suggested that both earlier trials and male gender have been associated with a greater likelihood of making a high-effort choice (Treadway et al. 2009).

Trials were considered ‘incomplete’ if the participant did not finish the button pressing in the allocated time. Participants were excluded from the analysis if they failed to complete ten or more trials on any one session. This was because we wished to exclude participants who did not carry out the task properly. The main outcome variable of the task was, on each trial, whether the participant made a low-effort or a high-effort choice.

It is possible that the speed at which a participant tapped affected choice behaviour. Hence, before the actual task, they were asked to press as fast as they could with their little finger in order to complete 30 and 100 presses; the time taken to make that number of presses (baseline button-pressing time) was recorded.

It is important to note that the EEfRT used here (as described above) was slightly different to the original EEfRT (Treadway et al. 2009) in a number of ways, the original version: (1) had more trials; (2) finished after a set amount of time, not a set amount of trials; (3) used the dominant index finger for the easy option; (4) had a continuous variation in money available to be won; and (5) gave participants 5 s to make their decision.

#### Drug history

Lifetime use was recorded as ‘yes’ or ‘no’. Current use (≥once per month) was recorded as ‘yes’ or ‘no’. We asked those who currently used how frequently (days/month) and how much (amount/session) they used.

#### Beck depression inventory (Beck et al. 1996)

This scale of depression severity consisted of 21 items that were rated for their frequency between 0 and 3 in the last week. Higher scores reflected greater depression severity.

#### Temporal experiences of pleasure scale (Gard et al. 2006)

This trait anhedonia scale consists of 18 items that are rated between 1 (very false for me) and 6 (very true for me). Two subscale scores are produced, namely anticipatory anhedonia and consummatory anhedonia. Higher scores reflect a greater ability to experience pleasure.

#### Snaith Hamilton pleasure scale (Snaith et al. 1995)

This state scale consists of 14 items that are rated between 0 (definitely agree) and 3 (definitely disagree), in terms of how a participant felt ‘right now’ (Powell et al. 2002). Higher scores reflect greater anhedonia.

#### Severity of dependence scale (Gossop et al. 1995)

This standard scale of drug dependence consists of five items that are rated between 0 and 4 in terms of frequency or difficulty with higher scores reflecting greater dependence severity.

#### ‘Stoned’ ratings

Participants gave ratings for stoned, right now from 0 (not at all) to 10 (extremely).

#### Drug administration

A Volcano Medic Vaporizer (Storz and Bickel, Tuttlingen, Germany) was used to vaporize Bedrocan cannabis (Veendan, the Netherlands). Across three occasions, we aimed to administer 8 mg THC (Cann-CBD), 8 mg THC + 10 mg CBD (Cann + CBD) and placebo (see Table 1). The amounts of THC and CBD that we aimed to administer were based broadly on previous THC/CBD vaporizer experiments (Bossong et al. 2009; Hindocha et al. 2015a) and Bedrocan product potencies (Brunt et al. 2014). This amount of THC is approximately equal to that which would be found in one quarter of a cannabis user’s joint, assuming that the cannabis has 10 % THC (Freeman et al. 2014). Furthermore, cannabis resin in the UK has approximately equal levels of THC and CBD (Hardwick and King 2008), which is similar to the ratio in the Cann + CBD in this study. Drugs were stored at −20 °C in foil-sealed pouches, then at ambient temperature prior to administration and then used within 6 months of purchase. Each dose was vaporized in two sequentially administered balloons to minimize residual cannabinoids. Participants were provided with video training at screening and inhaled at their own pace (each inhalation held for 8 s) until the balloon was empty. To maintain steady drug levels over time, participants received a 50 % top-up dose approximately 90 min later.

**Table 1:** Target doses of THC and CBD for Cann-CBD, Cann + CBD and placebo, and the weights of each cannabis type used to achieve them. THC dose and total weight were matched across sessions by adjusting the quantity of three cannabis varieties as shown below. All three cannabis types contained terpenoids, creating the distinctive smell of cannabis

	Cann-CBD	Cann + CBD	Placebo
Target dose	8 mg THC	8 mg THC + 10 mg CBD	N/A
Total weight	133.4 mg	133.4 mg	133.4 mg
‘Bedrobinol’ (12 % THC, <1 % CBD)	66.7 mg	N/A	N/A
‘Bediol’ (6 % THC, 7.5 % CBD)	N/A	133.4 mg	N/A
Placebo (derived from ‘Bedrocan’; <0.3 % THC, <1 % CBD)	66.7 mg	N/A	133.4 mg

### Procedure

Following telephone screening, participants attended a screening visit consisting of eligibility assessment, task training, drug history and trait questionnaires. Subsequently, they completed three testing sessions, on which they received Cann-CBD, Cann + CBD or placebo separated by a washout period of ≥7 days. Participants were asked to abstain from alcohol and any illicit drugs for ≥24 h before each testing session.

Testing sessions began with a urine sample to screen for pregnancy and to verify their recent self-reported drug use, assessed by 7 day Timeline Followback (Sobell and Sobell 1992). After drug administration, participants underwent MRI scanning for 1 h (data from the MRI section of the experiment will be reported elsewhere). Next, they received their top-up drug administration (approximately 90 min after the first) and began an approximately 90-min long battery of behavioural tasks. Participants completed ratings of stoned at five time points: (1) immediately before first drug administration (time ≈ 0 min), (2) immediately after first drug administration (time ≈ 5 min), (3) immediately before second drug administration (time ≈ 90 min), (4) immediately after second drug administration (time ≈ 95 min) and (5) end of the session (time ≈ 180 min).

### Statistical analyses

All analyses were carried out using IBM Statistical Package for Social Sciences (IBM SPSS version 22).

Stoned ratings were analysed using repeated measures ANOVA with two within-subject factors: drug (placebo, Cann-CBD, Cann + CBD) and time (1, 2, 3, 4, 5). Interactions were explored with Bonferroni corrected *t* tests. A repeated measures ANOVA with a within-subject factor of drug was used to analyse Snaith Hamilton pleasure scale (SHAPS) scores.

Generalized estimating equation (GEE) models were used to analyse the likelihood of participants making a high-effort choice. GEE models allow the outcome variable to be non-normally distributed with correlated residuals, which is a binary outcome in this case. GEE models allow parameters that vary on a trial-by-trial basis to be incorporated and they deal with missing data without excluding all of a participant’s data. Furthermore, these characteristic mean GEE models have more power to detect effects than general linear model approaches. The outcome measure was choice (high effort or low effort), modelled using a binary logistic distribution. We used an unstructured working correlation matrix.

Using the same approach as Treadway et al. (2009), we tested the effect of drug condition, and its interaction with task parameters on effort-related decision-making across four models. Each model included the standard predictors according to Treadway et al. (2009) (magnitude, probability, expected value, trial number, gender) and drug, with these additional predictors: no others (model 1), drug × magnitude (model 2), drug × probability (model 3) and drug × expected value (model 4). The categories of each factor were coded as follows: drug placebo = 0, Cann-CBD = 1 and Cann + CBD = 2; gender male = 0 and female = 1. Magnitude, probability, expected value and trial number were modelled as continuous predictors.

### Results

#### Demographics (Table 1 supplementary materials)^2^

Participants were aged 26.18 (SD = 7.13) years. On average, they smoked cannabis 8.06 (5.48) days per month, took 25.88 (33.73) days to smoke an 8 ounce (3.5 g) of cannabis and scored 1.13 (1.26) on the cannabis severity of dependence scale (SDS).

#### Drugs in urine

During the placebo session, THC was detected in eight and MDMA in one participants’ urine. During the Cann + CBD session, THC was detected in nine and PCP in one participants’ urine. During the Cann-CBD session, THC was detected in eight participants’ urine. No participants reported using any drugs within the last 24 h.

#### Stoned rating (Fig. 2)^3^

There was an interaction between time and drug (*F*_8,128_ = 20.296, *p* < 0.001), main effects of time (*F*_4,64_ = 82.443, *p* < 0.001) and drug (*F*_2,32_ = 56.154, *p* < 0.001). Ratings of stoned were the same at time 1 for all drug conditions. For every other time, both Cann-CBD and Cann + CBD conditions had greater ratings of stoned compared with placebo (all *p*s < 0.001) but did not differ from other. Stoned ratings did not differ between time 2 and time 4 for Cann-CBD or Cann + CBD (both *p*s = 1.000), demonstrating equivalent intoxication from the original dose and the 50 % top-up dose.

**Fig. 2:**
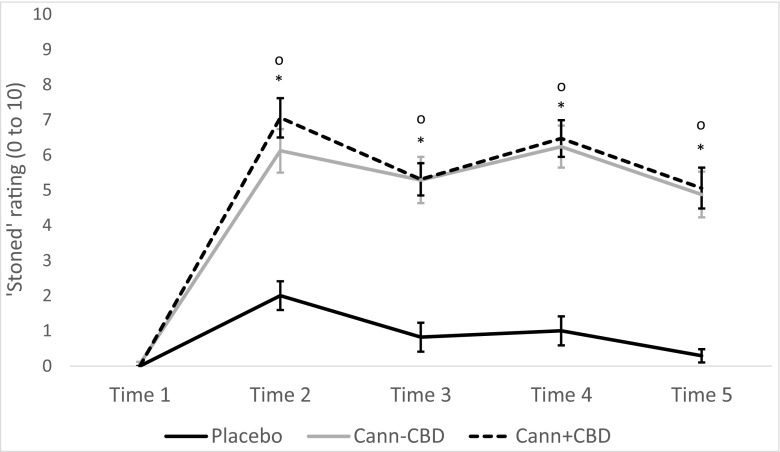
Mean (SE) scores for subjective ratings of ‘stoned’ and at five time points in study 1. Time 1 = immediately before first drug administration (0 min), time 2 = immediately after first drug administration (≈5 min), time 3 = immediately before second drug administration (≈90 mins), time 4 = immediately after second drug administration (≈95 min), time 5 = end of the session (≈180 mins). *Asterisk* indicates that Cann-CBD > placebo at *p* < 0.001. o indicates that Cann + CBD > placebo at *p* < 0.001

#### Effort Expenditure for Rewards Task

##### Baseline button-pressing time

There were no differences in baseline button-pressing time between any of the sessions.

##### Generalized estimating equation models (Table 2)^4^

**Fig. 3:**
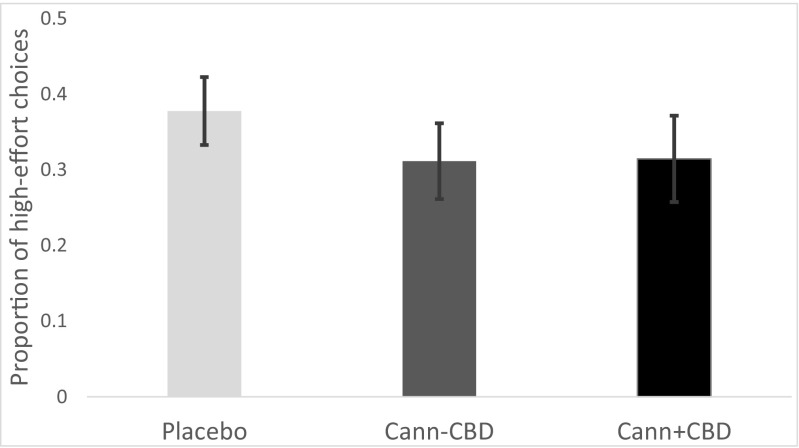
Mean (SE) numbers of high-effort choices made during each drug condition, collapsed across probability and magnitude, in study 1. There were 21 trials on each condition, so there were a maximum of 21 high-effort choices to be made. Error bars show standard error

Reward magnitude and probability both positively and significantly predicted making a high-effort choice in all models (*p*s < 0.01). The effect of EV approached significance in all models (*p*s < 0.1). As shown in model 1, Cann-CBD led to a lower likelihood of making a high-effort choice than placebo (*p* = 0.042), but there was no difference between Cann-CBD and Cann + CBD (Fig. 3). Model 3 found an interaction between drug and probability, such that Cann-CBD augmented the effect of probability on the likelihood of making a high-effort choice relative to placebo (*p* = 0.029). Model 4 found an interaction between drug and EV, such that Cann-CBD augmented the effect of EV on the likelihood of making a high-effort choice relative to both placebo (*p* = 0.014) and Cann + CBD (*p* = 0.006).

**Table 2:** GEE models for EEfRT from study 1

	Beta	SE	*p* value	Odds ratio	95 % CI OR
Model 1					
Magnitude	0.114	0.0315	<0.001	1.188	1.054, 1.193
Probability	0.172	0.0352	<0.001	1.121	1.109, 1.272
Expected value	0.134	0.0786	0.089	1.143	0.980, 1.333
Trial number	−0.008	0.0015	<0.001	0.992	0.989, 0.995
Gender	0.220	0.0720	0.002	1.246	1.082, 1.435
Placebo vs. Cann-CBD	**0.050**	**0.0247**	**0.042**	**1.051**	**1.002, 1.103**
Cann + CBD vs. Cann-CBD	**−0.001**	**0.0280**	**0.976**	**0.999**	**0.946, 1.056**
Model 2					
Magnitude	0.140	0.0405	0.001	1.151	1.063, 1.246
Probability	0.173	0.0353	<0.001	1.189	1.110, 1.274
Expected value	0.131	0.0786	0.095	1.140	0.978, 1.330
Trial number	−0.008	0.0015	<0.001	0.992	0.989, 0.995
Gender	0.220	0.0721	0.002	1.246	1.082, 1.435
Placebo vs. Cann-CBD	0.097	0.054	0.073	1.102	0.991, 1.224
Cann + CBD vs. Cann-CBD	0.055	0.0590	0.347	1.057	0.942, 1.187
(Placebo vs. Cann-CBD) × magnitude	**−0.033**	**0.0375**	**0.385**	**0.968**	**0.899, 1.042**
(Cann + CBD vs. Cann-CBD) × magnitude	**−0.039**	**0.0395**	**0.320**	**0.961**	**0.890, 1.039**
Model 3					
Magnitude	0.115	0.0313	<0.001	1.122	1.055, 1.193
Probability	0.206	0.0405	<0.001	1.229	1.135, 1.331
Expected value	0.131	0.0783	0.094	1.140	0.978, 1.329
Trial number	−0.008	0.0015	<0.001	0.992	0.989, 0.995
Gender	0.219	0.0716	0.002	1.245	1.082, 1.433
Placebo vs. Cann-CBD	0.123	0.0342	<0.001	1.131	1.057, 1.209
Cann + CBD vs. Cann-CBD	0.044	0.0356	0.212	1.045	0.975, 1.121
(Placebo vs. Cann-CBD) × probability	**−0.060**	**0.0276**	**0.029**	**0.942**	**0.892, 0.994**
(Cann + CBD vs. Cann-CBD) × probability	**−0.036**	**0.0199**	**0.073**	**0.965**	**0.928, 1.003**
Model 4					
Magnitude	0.117	0.0313	<0.001	1.124	1.057, 1.195
Probability	0.175	0.0352	<0.001	1.192	1.112, 1.277
Expected value	0.201	0.0793	0.011	1.223	1.047, 1.428
Trial number	−0.008	0.0015	<0.001	0.993	0.990, 0.995
Gender	0.219	0.0717	0.002	1.245	1.082, 1.433
Placebo vs. Cann-CBD	0.149	0.0387	<0.001	1.161	1.076, 1.253
Cann + CBD vs. Cann-CBD	0.078	0.0388	0.045	1.081	1.002, 1.166
(Placebo vs. Cann-CBD) × EV	**−0.121**	**0.0494**	**0.014**	**0.886**	**0.804, 0.976**
(Cann + CBD vs. Cann-CBD) × EV	**−0.093**	**0.0337**	**0.006**	**0.911**	**0.853, 0.973**

The likelihood of making a high-effort choice was predicted from each of the variables shown in the tables. Beta coefficients for each predictor term, standard errors, *p* values, odds ratios (ORs) and 95 % confidence intervals (CI) for these ORs are shown. The most important terms are in bold

The drug by probability interaction in model 3 was explored by carrying out GEE models within each level of probability. At low probability, Cann-CBD led to a *lower* likelihood of making high-effort choice than placebo (β = 0.188; SE = 0.0718; OR = 1.207; 95 % CI 1.049, 1.390). At medium and high probabilities, there were no significant differences on the likelihood of making a high-effort choice between Cann-CBD and placebo conditions.

Given that expected value = probability × magnitude, the drug by expected value interaction in model 4 was explored by replacing expected value by probability × magnitude terms and then carrying out GEE models within each level of probability (these models can be found in Table 2 supplementary materials^5^). At low probability, Cann-CBD led to a greater sensitivity to magnitude than Cann + CBD (β = 0.412; SE = 0.156; *p* = 0.008; OR = 1.510; 95 % CI 1.113, 2.048) and a marginally greater sensitivity to magnitude than placebo (β = 0.110; SE = 0.064; *p* = 0.086; OR = 1.117; 95 % CI 0.985, 1.267). However, at medium and high probabilities, there were no interactions between drug and magnitude. Therefore, the increase in sensitivity to expected value following Cann-CBD administration relative to Cann + CBD and placebo, found in model 4, can be attributed to an increase in sensitivity to magnitude changes at low probability following Cann-CBD, at least compared to Cann + CBD.

Results concerning the time to complete a trial and the number of completed trials within each drug condition are provided in the supplementary materials.

#### Snaith Hamilton pleasure scale

There was no effect of drug (*F*_2,32_ = 0.248, *p* = 0.782).

## Study 2

### Methods

#### Participants and design

Twenty cannabis-dependent individuals were compared with 20 controls, with eligibility criteria based on Morgan et al. (2012). Inclusion criteria for the cannabis-dependent participants were as follows: score ≥3 on the SDS for cannabis (indicative of dependence: Swift et al. 1998); smoke high-potency cannabis (‘skunk’) on 50 % or more of the occasions that they smoke cannabis; and score ≤2 on the SDS for all other drugs, except tobacco and alcohol. Participants in the control group were selected to match the cannabis-dependent group in terms of other (non-cannabis) drug use and had to score ≤2 on the SDS for all drugs, except tobacco and alcohol. Exclusion criteria for either group were as follows: currently seeking treatment for a mental health problem; current use of psychiatric medication; or diagnosis of alcohol dependence.

Participants were reimbursed £10/h. The study was approved by the UCL ethics committee, all participants provided written informed consent and the study was conducted in accordance with the Declaration of Helsinki.

### Assessments

The following measures were used as described in experiment 1: EEfRT, Beck depression inventory (BDI), temporal experiences of pleasure scale (TEPS), drug history and cannabis SDS.

#### Probabilistic Reward Task (Pizzagalli et al. 2005) (Fig. 4)

The task used abstract faces with two different lengths of mouth as the stimuli. The short mouth was 8 mm and the long mouth was 9 mm. The participant’s aim was to quickly determine whether the mouth was short or long. They sometimes won money (5p) if they correctly determined whether the mouth was long or short.

**Fig. 4:**
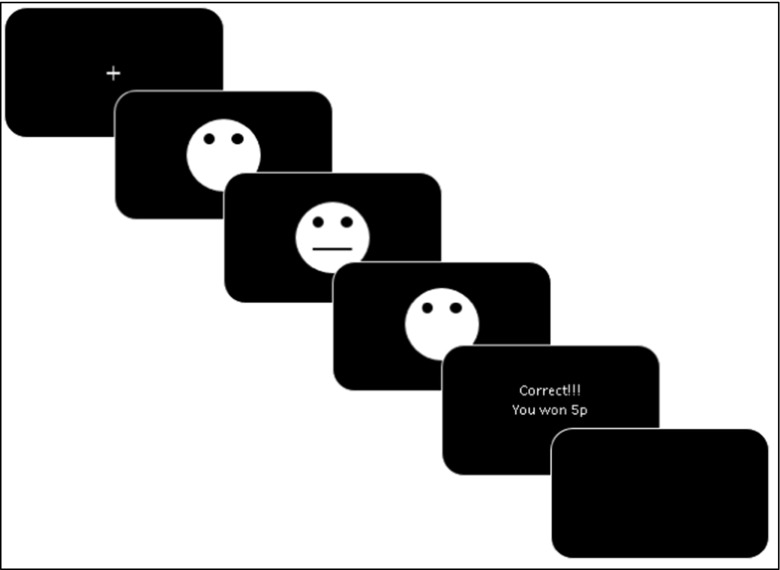
Diagrammatic representation of the Probabilistic Reward Task (Pizzagalli et al. 2005). (1) A fixation cross is shown for a jittered time (750 ms, 800 ms, 850 ms, or 900 ms), (2) a mouthless face is shown for 500 ms; (3) the mouth is added to the face for 97 ms; (4) the mouthless face is shown for 1500 ms or until the participant responds, stating they thought it is the long or short mouth; (5) feedback is given for 1500 ms; (6) a blank screen is shown for 2000 ms

The task composed two blocks of 100 trials. The trials were pseudo-randomized such that a maximum of 3 long or short mouths appeared consecutively. At the start of each trial, a fixation-cross was presented for a jittered time (750, 800, 850, or 900 ms). A mouthless face was then presented for 500 ms followed by the appearance of the mouth in the face for 97 ms. After the mouth disappeared, the mouthless face remained on the screen for 1500 ms or until the participant responded with the ‘v’ or ‘m’ key. The participant pressed the ‘v’ key if they thought the mouth was short and pressed the ‘m’ key if they thought the mouth was long. Subsequently, feedback was provided for 1500 ms, e.g. ‘Correct!!! You won 5p’ and then a blank screen was shown for 2000 ms.

Critically, one of stimuli/mouths (the ‘rich’ stimulus) was reinforced three times more frequently than the other stimulus/mouth (the ‘lean’ stimulus). Each block had 50 rich stimuli and 50 lean stimuli; 30 of the rich stimuli had the opportunity for reinforcement, while 10 of the lean stimuli had the opportunity for reinforcement. If a stimulus with the opportunity for reinforcement was not correctly identified, the next stimulus of that type (rich or lean) that was not going to be reinforced became a stimulus with the opportunity for reinforcement. This was to ensure that participants had similar numbers of reinforced rich and lean stimuli (ideally 30 and 10 respectively). Before the task began, participants were told that only some of the correct responses would be reinforced but they were not told that one of the stimuli was more likely to be reinforced than the other. An equal number of participants did the Probabilistic Reward Task (PRT) with the long mouth as the rich stimulus and the short mouth as the rich stimulus.

The differences between our task and the original PRT (Pizzagalli et al. 2005) were that, on our task, (1) participants won 5p on a successful trial, rather than 5 cents; (2) there were 200 trials split into two 100 trial blocks, rather than 300 trials split into 3 blocks; and (3) the mouth lengths were 8 and 9 mm, rather than 11.5 and 13 mm.

Trials and participants were excluded based on standard exclusionary criteria (Alexis Whitton, personal communication; Janes et al. 2015). Response bias (RB), discriminability, accuracy and reaction time were calculated as in previous papers.

Trials were excluded from analysis if the participant responded with a reaction time (RT) <100 ms or >1500 ms. Participants were excluded if, on either block, they had >20 % excluded trials, received reinforcement on <25 rich stimuli, received reinforcement on <6 lean stimuli, had <55 % accuracy for the rich stimulus and had <55 % accuracy overall (Alexis Whitton personal communication; Janes et al. 2015).

Response bias, which indexed a person’s bias towards the more frequently reinforced stimulus, was calculated using the following formula:

$$ \mathrm{Response}\ \mathrm{bias}=\frac{1}{2}* \log \frac{\mathrm{Rich}\_\mathrm{correct}*\mathrm{Lean}\_\mathrm{incorrect}\ }{\mathrm{Lean}\_\mathrm{correct}*\mathrm{Rich}\_\mathrm{incorrect}} $$

Discriminability, which indexed a person’s ability to differentiate the stimuli, was calculated using the following formula:

$$ \mathrm{Discriminability}=\frac{1}{2}* \log \frac{\mathrm{Rich}\_\mathrm{correct}*\mathrm{Lean}\_\mathrm{correct}\ }{\mathrm{Rich}\_\mathrm{incorrect}*\mathrm{Lean}\_\mathrm{incorrect}} $$

Rich_correct refers to the number of rich stimuli that was correctly identified. Lean_correct refers to the number of lean stimuli that was correctly identified. Rich_incorrect refers to the number of rich stimuli that was incorrectly identified. Lean_incorrect refers to the number of lean stimui that was incorrectly identified.

The task therefore produces one main outcome, response bias, and three other important outcomes: discriminability, accuracy and reaction time.

#### Spot-the-word (Baddeley et al. 1993)

This test, which correlates highly with premorbid verbal intelligence, consists of pairs of items: one’s a word and one’s a non-word. Participants decided which they thought was a real word.

### Procedure

Following telephone screening, participants completed one 2-h testing session. First, participants answered demographic and drug use questions, stated which drugs they had taken over the last 48 h and completed the spot-the-word test. Subsequently, they completed the EEfRT, the BDI, the TEPS and the PRT and provided a urine sample. Participants also completed three other cognitive tasks and questionnaires concerning psychosis-like symptoms, which will be reported elsewhere.

### Statistical analyses

All analyses were carried out using IBM Statistical Package for Social Sciences (IBM SPSS version 22). Where appropriate, errors were checked for normality, unbiasedness and homoscedasticity using inspection of histograms and Levene’s test. Non-parametric tests were used when data did not meet the above assumptions, and a suitable test was available.

Analysis of the EEfRT was conducted in the same way as in study 1. We tested whether group and its interactions with task parameters affected the likelihood of making a high-effort choice across four models. Each model included the standard predictors (see above) and group, with the additional predictors: no others (model 1), group × magnitude (model 2), group × probability (model 3) and group × expected value (model 4). Each model also included BDI, average number of cigarettes/day and baseline button-pressing time because of group differences on these variables. The models were also run without these three extra predictors to see if it affected the pattern of results.

For the PRT, RB and discriminability were analysed with mixed ANOVAs with a between-subject factor of group (controls, cannabis) and within-subject factors of block (1, 2). Accuracy and RTs were analysed in the same way but with an extra within-subject factor of stimulus (rich, lean). ANCOVAs were used to investigate whether inclusion of BDI and average number of cigarettes/day affected results.

Correlations were computed for composite RB (averaged across blocks 1 and 2) and ∆RB (change between blocks 1 and 2) with BDI, average number of cigarettes/day (which includes those who do not smoke and those who do not smoke every day) and cannabis-SDS in each group separately.

### Results

#### Demographics (Table 3 supplementary materials)

The groups did not differ in gender, age, highest level of education achieved or any measure of illicit drug use. However, compared with the controls, the cannabis group, on average, had a higher BDI score^6^ (*t*_38_ = 2.932, *p* = 0.006), a lower spot-the-word score (*t*_38_ = 2.585, *p* = 0.014) and smoked more cigarettes/day (*t*_38_ = 4.411, *p* < 0.001).

All but two of the cannabis group smoked cannabis every day; one participant smoked approximately 22 days per month and another smoked approximately 12 days per month. The cannabis group smoked an average of 1.49 g (1.41) per session and had an average cannabis SDS score of 7.30 (3.39). Eight controls smoked cannabis at least once per month, with an average of 3.94 days (1.78) per month and an average of 0.31 g (0.28) per session. None of the controls scored >0 on the cannabis SDS.

#### Recent drug use

No participants reported using cannabis, alcohol or any other illicit drug within 12 h of testing.

In the control group, there were positive urine tests for THC (*n* = 4), benzodiazepines (*n* = 2), buprenorphine (*n* = 2), cocaine (*n* = 1), PCP (*n* = 1) and opioids (*n* = 1).^7^ In the cannabis group, there were positive urine tests for THC (*n* = 19), cocaine (*n* = 2) and opioids (*n* = 2).

#### Effort Expenditure for Rewards Task

##### Baseline button-pressing time

The controls were faster than the cannabis-dependent participants to complete 30 and 100 button presses (*t*_37_ = 3.113, *p* = 0.004). As a result, baseline button-pressing time was included in the GEE models.

##### Generalized estimating equation models (Table 4 supplementary materials).^8^

Reward magnitude and probability positively predicted making a high-effort choice in all models (*p*s < 0.05), and expected value did so in all but one of the models (*p*s < 0.05). Participants were less likely to make a high-effort choice as the task went on, as demonstrated by the negative effect of trial number (*p*s < 0.001). However, there was no overall difference in motivation between the groups and no interactions between group and magnitude, probability or expected value. The pattern of these results did not change when we removed baseline button-pressing, BDI and average number of cigarettes/day from the models.

#### Probabilistic Reward Task

##### Response bias (Fig. 5)^9^

Repeated measures ANOVA revealed a trend interaction between group and block (*F*_1,27_ = 3.579, *p* = 0.069), a main effect of group, indicating lower RB in the cannabis group (*F*_1,27_ = 8.531, *p* = 0.007), and a trend effect of block, reflecting increased RB from blocks 1 to 2 (*F*_1,27_ = 2.978, *p* = 0.096).

**Fig. 5:**
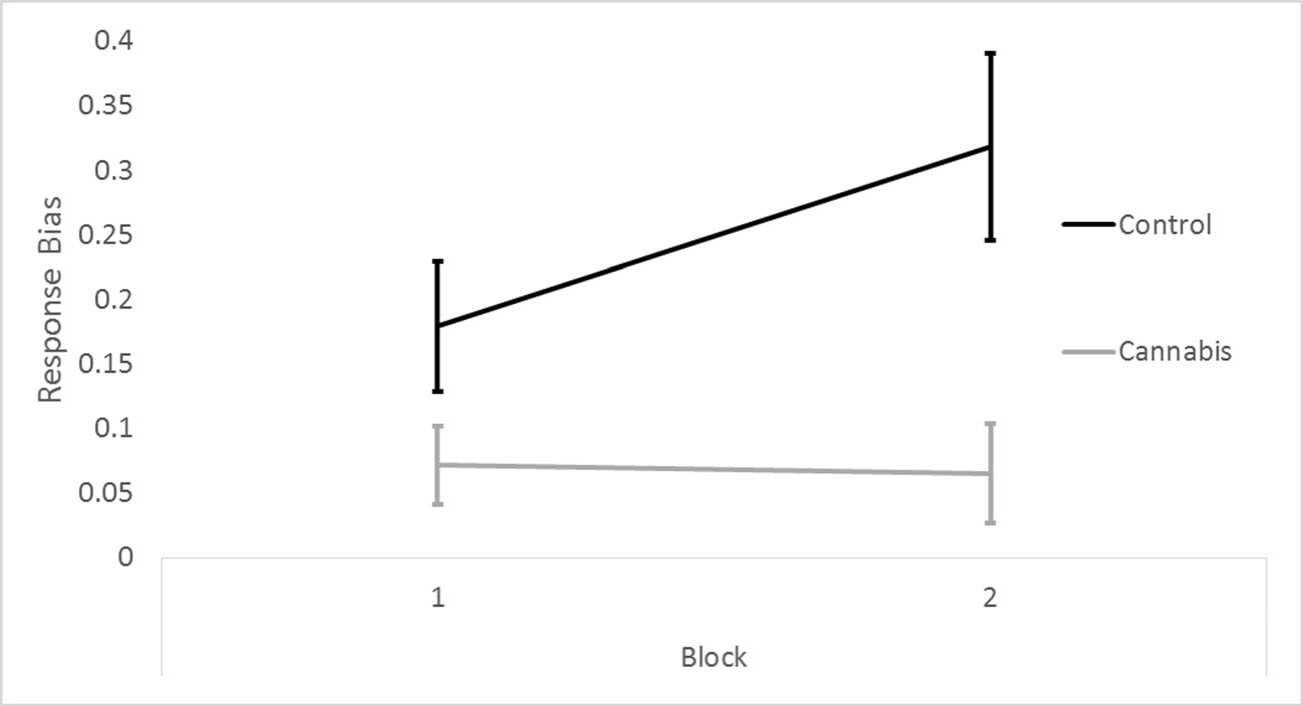
Means (SE) for response bias on the PRT for the control participants (control) and the cannabis-dependent (cannabis) participants, on blocks 1 and 2, in study 2. Out of 40 participants, 11 were excluded; 5 were from the drug-using control group and 6 were from the cannabis dependent group. Error bars show standard error

Exploration of the trend group by block interaction showed that RB increased from blocks 1 to 2 in controls (*t*_14_ = 2.604, *p* = 0.015) but not cannabis users (*t*_13_ = 0.109, *p* = 0.909). Furthermore, RB was significantly greater in controls than cannabis users during block 2 (*t*_25_ = 3.00, *p* = 0.005) but only marginally so in block 1 (*t*_25_ = 1.831, *p* = 0.082).

All of these effects were lost when BDI and average number of cigs/day were included as covariates. There was a trend main effect of BDI (*F*_1,25_ = 3.464, *p* = 0.075) and no effect of cigs/day.

The pattern of results did not change if all of the participants were included in the analysis.

#### Discriminability

There was a trend towards an effect of block, with greater discriminability in block 2 compared with block 1 (*F*_1,27_ = 3.605, *p* = 0.068), no effect of group nor an interaction between the two. The trend effect of block was lost when BDI and average number of cigs/day were included as covariates.

#### Accuracy

There was an interaction between group and stimulus (*F*_1,27_ = 8.723, *p* = 0.006) and a main effect of stimulus, with greater accuracy for the rich stimulus (*F*_1,27_ = 28.109, *p* < 0.001). No other effects or interactions were significant.

Exploration of the interaction showed that the controls had greater accuracy for the rich stimulus compared with the lean stimulus (*t*_14_ = 5.941, *p* < 0.001) while the cannabis group did not. The main effect of stimulus remained after including the covariates, but the interaction between group and stimulus was lost.

#### Reaction time

There was a main effect of stimulus, with a faster response to the rich stimulus compared with the lean stimulus (*F*_1,27_ = 7.684 *p* = 0.010). No other effects or interactions were significant. This effect was unchanged when including the covariates.

#### Correlations

Within each group separately, none of the correlations examined reached significance.

## Discussion

Historically, cannabis use has been linked to amotivation (McGlothlin and West 1968), and cannabis dependence is theoretically associated with non-drug reward processing deficits (Goldstein and Volkow 2011), although empirical evidence for these claims is lacking. To the authors’ knowledge, this report is the first to examine the acute effects of different cannabinoids on effort-related decision-making and to investigate associations between cannabis dependence and effort-related decision-making and reward learning.

In study 1, acute administration of cannabis without CBD (Cann-CBD) reduced the overall likelihood of making high-effort choices (i.e. motivation) for monetary reward compared with placebo. Contrary to our hypothesis, this effect was not, overall, attenuated by cannabis with CBD (Cann + CBD). However, Cann-CBD increased sensitivity to expected value of the monetary outcomes, relative to both placebo (OR = 1.129) and Cann + CBD (OR = 1.092); this was due to magnitude having a greater effect on behaviour in the Cann-CBD condition at low probability. These data therefore suggest that acute cannabis administration can lead to transient amotivation and they provide some evidence that CBD partially moderates the effects of THC on motivation, via altering the way THC interacts with expected value. In study 2, no relationship between cannabis dependence and effort-related decision-making emerged. However, cannabis-dependent participants had overall weaker reward learning than the controls, and the cannabis-dependent participants also failed to improve their response bias between blocks. Due to other group differences and the nature of the study, it is hard to conclude whether these effects were driven by cannabis dependence or confounding variables, such as depression.

### Acute cannabis and motivation

Despite enduring beliefs that cannabis acutely reduces motivation, we are aware of only one controlled study which used a work-for-reward design (Cherek et al. 2002), and they had a sample of five participants. Some older work had suggested null (Mello and Mendelson 1985; Mendelson et al. 1976) or pro-motivational (Foltin et al. 1990) effects of acute cannabis; however, these studies were not well controlled or did not provide a clear reward respectively. Here, the results provide evidence to support this hypothesis using a task that has previously demonstrated sensitivity to anhedonia, major depressive disorder and dopaminergic function ( Treadway et al. 2009, 2012a, b; Wardle et al. 2011). In the first model, placebo, relative to Cann-CBD, was a significant, positive predictor of the likelihood of making a high-effort choice. Hence, the administration of Cann-CBD reduced motivation for monetary reward, and this supports a transient amotivational effect. It is difficult to speculate on the pharmacology underlying this effect. THC may boost dopamine release (Bossong et al. 2009), which would be expected to enhance motivation, but we found the opposite. The endocannabinoid system’s role in motivation must be more clearly elucidated before attempting to explain in detail THC’s amotivational effects, but this result at least suggests that functioning of CB1 receptors is important in effort-related decision-making.

Although CBD has been shown to shield individuals against some of the negative effects of THC (Englund et al. 2013; Hindocha et al. 2015a; Morgan et al. 2010a), the overall difference between Cann-CBD and Cann + CBD was null in the first model. There is thus no evidence that cannabidiol reduced the overall amotivational effects of THC. It may be the case that a higher dose of cannabidiol or a different time of administration relative to THC is needed to produce a stronger pro-motivational effect.

However, Cann-CBD influenced the effects of expected value on effort-related decision-making differently to Cann + CBD. Expected value refers to the multiplication of the outcome value with the probability of receiving the outcome, so it represents how good an option is and how much it is worth. According to model 4, expected value increased the likelihood of making a high-effort choice more following administration of Cann-CBD than placebo and Cann + CBD. This implies that CBD affected the way people made decisions about different effortful outcomes. Further exploration showed that these drug by expected value interactions were at least partially due to Cann-CBD, significantly and marginally increasing sensitivity to magnitude on low probability trials relative to Cann + CBD and placebo respectively. In other words, at low probability, magnitude had a larger effect on behaviour following Cann-CBD than Cann + CBD and, to some extent, placebo. These results could suggest that the presence of CBD attenuated THC’s effects on the processing of expected value, such that Cann + CBD was more similar to placebo than Cann-CBD, in this regard. Alternatively, one could conclude that the presence of CBD made it less like placebo: Cann-CBD augmented the effect of expected value more than Cann + CBD and placebo, so this means that as expected value increased, Cann-CBD somewhat recovered from its amotivational effects, while Cann + CBD did not. Therefore, CBD’s role in effort-related decision-making is slightly ambiguous. Replications of this study are needed before any conclusive remarks about CBD’s motivational qualities are made.

Importantly, becoming stoned is a major motivator for cannabis use and it is noteworthy that CBD did not compromise this desired effect of THC, consistent with previous findings (Haney et al. 2015; Hindocha et al. 2015a). The lack of CBD’s effect on stoned ratings may be important in harm reduction messages if users wish to maintain the degree to which they feel subjective effects, while potentially reducing some of the harmful consequences of THC (Curran et al. 2016).

### Cannabis dependence and reward processing impairments

No association emerged between cannabis dependence and effort-related decision-making. The results are concordant with previous survey-based research which have failed to find a relationship between long-term cannabis use and self-reported motivation (Barnwell et al. 2006; Musty and Kaback 1995). Thus, these results imply that cannabis acutely but not chronically alters effort-related decision-making. However, given the cross-sectional nature of the study, the results should be interpreted cautiously. A large, longitudinal study that records frequency of cannabis use, type of cannabis used and different aspects of motivation is needed to more thoroughly address the question of how chronic use might relate to amotivation.

Similar to the associations with depression (Pizzagalli et al. 2008) and nicotine withdrawal (Pergadia et al. 2014), we demonstrated that cannabis dependence (with >12 h of abstinence) was associated with reduced reward learning compared with non-dependent, drug-using controls. Not only did the cannabis-dependent individuals have an overall reduced response bias, but they also did not improve their response bias between blocks, as is usually seen in healthy controls (Pizzagalli et al. 2005), although the group by block interaction was only a trend.

Drug addiction has been associated with deficits in non-drug reward processing (Goldstein and Volkow 2002; Lubman et al. 2009) and anhedonia (Garfield et al. 2014; Hatzigiakoumis et al. 2011; Leventhal et al. 2008). Given cannabis’s putative effects on reward circuitry (Bloomfield et al. 2014a; Maldonado et al. 2006) and the depressive effects of cannabis withdrawal (Budney and Hughes 2006), our finding concerning reduced reward learning was expected. Whether this reward deficiency was a consequence of chronic cannabis, a predisposing factor for cannabis use, other factors or a combination of these remain to be seen and will require longitudinal studies. Whatever the causal relationships, a reduced capacity to direct behaviour towards more reinforced stimuli is an important finding as it may contribute to reduced subjective wellbeing and could negatively impact treatment success, as seen in depression (Vrieze et al. 2013).

Although the groups were very similar in terms of other illicit drug use, age, gender and educational achievement, they did differ significantly in depression levels and tobacco use. This is not surprising, given that depression and tobacco use are positively associated with cannabis dependence (Hindocha et al. 2015b). We found that when these factors were included as covariates, the effects of group and block were lost. Given this result and the strong relationship between depression and reward responsiveness on the PRT (Pizzagalli et al. 2005, 2008), as well as emerging evidence that tobacco use and nicotine withdrawal affect task behaviour (Janes et al. 2015; Liverant et al. 2014; Pergadia et al. 2014), drawing any conclusions about specific relationships between cannabis use, tobacco use, depression and reward learning is difficult. However, just because the effect of group was lost when depression and cigarette smoking were included as covariates, this does not mean that cannabis dependence is not associated with reduced reward learning. As a relatively large amount of variance was shared between group and tobacco use (approximately 30 %) and depression (approximately 20 %), covarying for these variables is not statistically optimal and may be considered inappropriate (Miller and Chapman 2001). Future case-control studies should therefore aim to match groups on depression and cigarette smoking.

It is also important to note that we did not include a healthy, non-drug-using control group in this study. Cannabis-dependent individuals may well have impaired effort-related decision-making and reward learning relative to this alternative group. Indeed, the drug-using controls in study 2 may even have some reward-processing deficits relative to healthy controls. However, our choice of control group provided the most conservative test for reward-processing deficits attributable to chronic cannabis use.

## Strengths and limitations

Study 1 was a placebo-controlled, double-blind experiment and so provides strong evidence for cannabis *causing* transient amotivation. To the authors’ knowledge, this is the first time this has been shown in an adequately powered study (they are consistent with a previous study (Cherek et al. 2002) with a sample size of five). Furthermore, the investigation of CBD was highly novel. We found preliminary evidence that it can moderate the effects of THC on effort-related decision-making. The drug administration protocol was effective as stoned ratings were similar immediately after the first and second doses. Although cannabis-dependent participants were more depressed and smoked more cigarettes than drug-using controls in study 2, they were well matched on all other demographic variables, including other drug use, which is a key strength.

One important limitation of both studies was that there were positive drug urine test results for various participants, and residual drug effects could have affected task performance. Furthermore, the criteria for inclusion in the cannabis dependent group could have been improved by carrying out interviews to assess DSM cannabis dependence/use disorder. Although all cannabis-dependent participants smoked skunk on ≥50 % times they smoked cannabis, we did not actually assess preference of cannabis type in the cannabis-dependent group and so we may have missed out on reward-processing differences between skunk-preferring and hash-preferring participants.

While these two studies have addressed the acute effects of cannabis on and association of cannabis dependence with reward processing, we only employed one type of reward. Money, as a secondary reward, activates somewhat different brain regions compared with primary rewards (Sescousse et al. 2010) and may also be considered a way of buying drugs, rather than being seen as a reward in itself. Future studies should investigate reward processing of a variety of rewards, including cannabis itself, so that comparisons between drug and non-drug reward processing can be made (Lawn et al. 2015). Moreover, although urinalysis was conducted in both experiments, we were not able to relate task performance to quantitative indices of cannabinoid metabolites, which could have improved our ability to infer acute and chronic effects of THC and CBD (Morgan et al. 2012). Finally, study 2 could obviously have been improved if depression and cigarette smoking were not different between the groups.

## Conclusions

In conclusion, cannabis without CBD led to an overall reduction in motivation as evidenced by a lower likelihood of making a high-effort choice to earn monetary reward. Cannabis with CBD did not appear to reduce this effect but did moderate THC’s effects on expected value to some extent. Cannabis dependence was associated with preserved motivation and impaired reward learning. However, given the observational nature of the data and the confounding group differences, it is difficult to ascertain what caused the impaired reward learning. In summary, these results support a transient amotivational syndrome caused by acute cannabis administration but do not support a chronic amotivational syndrome associated with cannabis dependence. Future research should employ large, longitudinal designs to better probe reward processing impairments in long-term cannabis users.

## Electronic supplementary material

ESM 1 (DOCX 35.3 kb)

## Footnotes

1 As 17 is not divisible by 3, the Latin square was not completed with equal numbers of participants in each treatment order.

2 Data was missing for one participant for BDI, TEPS and drug history

3 One participant missed a rating at time 3 on the Cann + CBD session; this was imputed from the group mean.

4 We excluded one participant for failing to complete 13 and 14 trials on two of his sessions, thus he clearly did not complete the task as instructed.

5 We thank an anonymous reviewer for this suggestion.

6 One control’s BDI score was missing so it was imputed from the group mean

7 One control’s urine test results were missing

8 One cannabis-dependent participant was excluded because they failed to complete 16 trials.

9 Eleven out of 40 participants were excluded due to not meeting task criteria, 5 were drug-using controls and 6 were cannabis-dependent participants.
