# Supplementary material for: Acute and chronic effects of cannabinoids on effort-related decision-making and reward learning: an evaluation of the cannabis ‘amotivational’ hypotheses
Source: Psychopharmacology (Berl). 2016 Sep 2;233(19):3537–52. doi: 10.1007/s00213-016-4383-x (PMC5021728; doi:10.1007/s00213-016-4383-x)
Supplement: Supplementary file 1 — (DOCX 35.3 kb) [file 213_2016_4383_MOESM1_ESM.docx]

**Psychopharmacology**

**Electronic Supplementary Material 1**

Acute and chronic effects of cannabinoids on effort-related decision-making and reward learning: an evaluation of the cannabis ‘amotivational’ hypotheses.

Will Lawn^1^, Tom P Freeman^1^, Rebecca A Pope^1^, Alyssa Joye^1^, Lisa Harvey^1^, Chandni Hindocha^1^, Claire Mokrysz^1^, Abigail Moss^1^, Matthew B Wall^1,2,3^, Michael AP Bloomfield^1,4,5^, Ravi K Das^1^, Celia JA Morgan^1,6^, David J Nutt^7^, H Valerie Curran^1^

^1^Clinical Psychopharmacology Unit, University College London, Gower Street, London, UK.

**^2^**Imanova Ltd, Burlington Danes Building, Imperial College London, Hammersmith Hospital, Du Cane Road, London, UK.

^3^Division of Brain Sciences, Imperial College London, London, UK.

**^4^**Psychiatric Imaging Group, Medical Research Council Clinical Sciences Centre, Hammersmith Hospital, London, UK.

^5^Division of Psychiatry, University College London, Maple House, London, UK.

^6^Centre for Clinical Psychopharmacology, University of Exeter, Exeter, UK.

^7^Neuropsychopharmacology Unit, Division of Experimental Medicine, Imperial College London, Burlington Danes Building, Du Cane Road, London, UK.

**Results study 1**

*Time to complete an easy trial and a hard trial*

An ANOVA with within-subjects factors of drug (placebo, Cann-CBD and Cann+CBD) and choice (low-effort, high-effort) was conducted. There was no effect of drug or interaction between drug and choice. Hence, the drug type did not significantly affect time to complete a trial, and thus button-pressing speed during the task.

*Completion of trials*

Out of a total of 21 trials, on placebo an average of 20.15 (SD=1.51) trials were completed, on Cann+CBD an average of 19.47 (SD=2.33) trials were completed, and on Cann-CBD an average of 19.80 (SD=1.86) trials were completed. There was no significant effect of drug on the number of trials completed.

**Results study 2**

*Time to complete an easy trial and a hard trial*

An ANOVA with a between-subjects factor of group (drug-using control and cannabis dependent) and a within-subjects factor of choice (low-effort and high-effort) was conducted. There was no significant effect of group or interaction between group and choice. Hence, there was no difference between drug-using controls and cannabis dependent participants on time to complete a trial, and thus button-pressing speed during the task.

*Completion of trials*

Out of 21 trials, the drug-using controls completed an average of 20.90 (SD=0.30) trials and the cannabis dependent participants completed an average of 20.21 (SD=0.39) trials. There was no significant effect of group or an interaction between group and choice on the number of trials completed.

**Tables**

*Table 1 Means (S.D.) and frequencies for demographic data and drug use for participants in study 1. Data was missing for one participant for BDI, TEPS and drugs history.*

|  | Participants |
| --- | --- |
| Age | 26.18 (7.13) |
| Gender (m/f) | 8/9 |
| BDI | 3.38 (3.12) |
| TEPS consummatory | 43.50 (5.61) |
| TEPS anticipatory | 42.06 (4.85) |
| TEPS total | 86.56 (9.30) |
| Cannabis SDS | 1.13 (1.26) |
| Alcohol ever used (y/n) | 16/0 |
| Alcohol use now (y/n) | 16/0 |
| Alcohol days per month | 10.81 (4.86) |
| Alcohol units/session | 5.93 (2.08) |
| Amphetamine ever used (y/n) | 8/8 |
| Amphetamine use now (y/n) | 0/16 |
| Amphetamine days per month | NA |
| Amphetamine grams/session | NA |
| Cannabis ever used (y/n) | 16/0 |
| Cannabis use now (y/n) | 16/0 |
| Cannabis days per month | 8.06 (5.48) |
| Cannabis days to smoke an 8th | 25.88 (33.73) |
| Cocaine ever used (y/n) | 11/5 |
| Cocaine use now (y/n) | 3/13 |
| Cocaine days per month | 1.0 (0.0) |
| Cocaine grams/session | 0.5 (0.0) |
| Heroin ever used (y/n) | 0/16 |
| Heroin use now (y/n) | 0/16 |
| Heroin days per month | NA |
| Heroin grams/session | NA |
| Ketamine ever used (y/n) | 10/6 |
| Ketamine use now (y/n) | 2/14 |
| Ketamine days per month | 1.50 (0.71) |
| Ketamine grams/session | 0.75 (0.35) |
| Mephedrone ever used (y/n) | 7/9 |
| Mephedrone use now (y/n) | 0/16 |
| Mephedrone days per month | NA |
| Mephedrone grams/session | NA |
| MDMA ever used (y/n) | 14/2 |
| MDMA use now (y/n) | 6/10 |
| MDMA days per month | 1.50 (0.84) |
| MDMA grams/session | 0.31 (0.19) |
| Tobacco ever used (y/n) | 15/1 |
| Tobacco use now (y/n) | 15/1 |
| Tobacco days per month | 11.30 (10.27) |
| Tobacco cigs/day (when smoking) | 3.63 (3.62) |
| Tobacco average cigs/day | 2.16 (3.48) |

*Table 2 GEE Models for EEfRT from study 1. The likelihood of making a high-effort choice was predicted from each of the predictors shown in the tables below. The models were calculated at different levels of probability separately: model 1: low probability (12%), model 2: medium probability (50%), model 3: high probability (88%). This was to investigate the drug X expected value interaction which was found to be significant. Beta coefficients for each predictor term, standard errors, p-values, odds ratios (OR) and 95% confidence intervals (CI) for these ORs are shown. The most important terms are in bold.*

Model 1

|  | Beta | S.E. | p | Odds Ratio | 95% CI OR |
| --- | --- | --- | --- | --- | --- |
| Magnitude | 0.058 | 0.0310 | 0.062 | 1.059 | 0.997, 1.126 |
| Trial number | -0.011 | 0.0030 | <0.001 | 0.989 | 0.983, 0.995 |
| Gender | 1.142 | 0.427 | 0.007 | 3.133 | 1.357, 7.232 |
| Placebo vs. Cann-CBD | 0.032 | 0.103 | 0.752 | 1.033 | 0.845, 1.263 |
| Cann+CBD vs. Cann-CBD | -0.463 | 0.176 | 0.008 | 0.630 | 0.446, 0.889 |
| (Placebo vs. Cann-CBD) X magnitude | **0.110** | **0.064** | **0.086** | **1.117** | **0.985, 1.267** |
| (Cann+CBD vs. Cann-CBD) X magnitude | **0.412** | **0.156** | **0.008** | **1.510** | **1.113, 2.048** |

Model 2

|  | Beta | S.E. | p | Odds Ratio | 95% CI OR |
| --- | --- | --- | --- | --- | --- |
| Magnitude | 0.247 | 0.0603 | <0.001 | 1.280 | 1.137, 1.440 |
| Trial number | -0.013 | 0.0028 | <0.001 | 0.987 | 0.982, 0.993 |
| Gender | 0.462 | 0.250 | 0.064 | 0.064 | 0.973, 2.589 |
| Placebo vs. Cann-CBD | -0.120 | 0.206 | 0.560 | 0.887 | 0.592, 1.328 |
| Cann+CBD vs. Cann-CBD | 0.094 | 0.130 | 0.472 | 1.098 | 0.851, 1.418 |
| (Placebo vs. Cann-CBD) X magnitude | **0.089** | **0.143** | **0.533** | **1.093** | **0.826, 1.448** |
| (Cann+CBD vs. Cann-CBD) X magnitude | **-0.071** | **0.101** | **0.481** | **0.931** | **0.765, 1.135** |

Model 3

|  | Beta | S.E. | p | Odds Ratio | 95% CI OR |
| --- | --- | --- | --- | --- | --- |
| Magnitude | 0.416 | 0.0888 | <0.001 | 1.517 | 1.274, 1.805 |
| Trial number | -0.009 | 0.0042 | 0.024 | 0.991 | 0.982, 0.999 |
| Gender | 0.366 | 0.1262 | 0.004 | 1.443 | 1.126, 1.847 |
| Placebo vs. Cann-CBD | 0.261 | 0.195 | 0.181 | 1.298 | 0.885, 1.903 |
| Cann+CBD vs. Cann-CBD | 0.113 | 0.190 | 0.550 | 1.120 | 0.772, 1.624 |
| (Placebo vs. Cann-CBD) X magnitude | **-0.173** | **1.395** | **0.215** | **0.841** | **0.640, 1.106** |
| (Cann+CBD vs. Cann-CBD) X magnitude | **-0.098** | **0.125** | **0.432** | **0.907** | **0.710, 1.158** |

*Table 3 Demographic details and drug history for non-dependent, drug-using controls and cannabis-dependent participants in study 2. *p<0.05, **p<0.01, ***p<0.001*

|  | Control | Cannabis |
| --- | --- | --- |
| Age | 27.25 (6.80) | 27.75 (7.31) |
| Gender (f/m) | 6/14 | 7/13 |
| Highest education level (GCSE/NVQ-BTEC-Diploma/A-Levels/Undergraduate Degree/Postgraduate Degree) | 2/2/5/9/2 | 5/3/1/10/1 |
| BDI** | 5.32 (5.41) | 12.20 (9.00) |
| TEPS consummatory | 39.25 (6.21) | 35.80 (6.70) |
| TEPS anticipatory | 46.45 (6.69) | 46.10 (7.45) |
| TEPS total | 86.10 (11.76) | 81.90 (12.63) |
| Spot-the-word* | 48.30 (3.36) | 45.35 (3.84) |
| Cannabis SDS*** | 0 | 7.30 (3.39) |
| Alcohol ever used (y/n) | 20/0 | 20/0 |
| Alcohol use now (y/n) | 20/0 | 17/3 |
| Alcohol days per month | 14.11 (7.49) | 10.97 (8.64) |
| Alcohol units/session | 7.75 (4.61) | 7.72 (4.26) |
| Amphetamine ever used (y/n) | 9/11 | 9/11 |
| Amphetamine use now (y/n) | 0/20 | 1/19 |
| Amphetamine days per month | NA | 1 |
| Amphetamine grams/session | NA | 0.1 |
| Benzodiazepines ever used (y/n) | 9/11 | 10/10 |
| Benzodiazepines use now (y/n) | 1/19 | 3/17 |
| Benzodiazepines days per month | 2 | 2.83 (1.04) |
| Benzodiazepines tablets per session | 1 | 1.25 (1.06) |
| Cannabis ever used (y/n) | 20/0 | 20/0 |
| Cannabis use now (y/n)*** | 8/12 | 20/0 |
| Cannabis days per month*** | 3.94 (1.78) | 28.19 (4.74) |
| Cannabis grams/session*** | 0.31 (0.28) | 1.49 (1.41) |
| Cocaine ever used (y/n) | 16/4 | 14/6 |
| Cocaine use now (y/n) | 8/12 | 4/16 |
| Cocaine days per month | 1.88 (0.84) | 3.00 (1.41) |
| Cocaine grams/session | 0.59 (0.33) | 0.75 (0.29) |
| Hallucinogens use now (y/n) | 1/19 | 1/19 |
| Hallucinogens days per month | 1 | 1 |
| Heroin ever used (y/n) | 2/18 | 3/17 |
| Heroin use now | 0/20 | 0/20 |
| Heroin days per month | NA | NA |
| MDMA ever used (y/n) | 18/2 | 16/4 |
| MDMA use now (y/n) | 7/13 | 5/15 |
| MDMA days per month | 1.43 (0.787) | 1.40 (0.548) |
| MDMA grams/session | 0.34 (0.33) | 0.44 (0.13) |
| Mephedrone ever used (y/n) | 7/13 | 6/14 |
| Mephedrone use now (y/n) | 0/20 | 0/20 |
| Mephedrone days per month | NA | NA |
| Mephedrone grams/session | NA | NA |
| Tobacco ever used (y/n) | 18/2 | 0/20 |
| Tobacco use now (y/n)** | 9/11 | 19/1 |
| Tobacco days per month* | 18.39 (12.95) | 29.26 (3.21) |
| Tobacco cigs/day (when smoking)* | 1.14 (2.53) | 7.55 (5.19) |
| Tobacco average cigs/day* | 1.43 (2.54) | 7.01 (5.38) |

*Table 4 GEE Models for EEfRT from study 2. The likelihood of making a high-effort choice was predicted from each of the predictors shown in the tables below. Beta coefficients for each predictor term, their standard errors, associated p-values, odds ratios (OR) and 95% confidence intervals (CI) for these ORs are shown. Av-Cigs/Day = average number of cigarettes smoked per day. The most important terms are in bold.*

Model 1

|  | Beta | S.E. | p | Odds Ratio | 95% CI OR |
| --- | --- | --- | --- | --- | --- |
| Magnitude | 0.236 | 0.0845 | 0.005 | 1.266 | 1.073, 1.494 |
| Probability | 0.278 | 0.0814 | 0.001 | 1.320 | 1.126, 1.549 |
| Expected Value | 0.278 | 0.1132 | 0.014 | 1.321 | 1.058, 1.649 |
| Trial Number | -0.015 | 0.0028 | <0.001 | 0.985 | 0.980, 0.990 |
| Gender | 0.125 | 0.0909 | 0.169 | 1.133 | 0.948, 1.354 |
| BDI | -0.006 | 0.0048 | 0.232 | 0.994 | 0.985, 1.004 |
| Av-Cigs/Day | -0.007 | 0.0069 | 0.297 | 0.993 | 0.979, 1.006 |
| Baseline button-pressing time | 0.011 | 0.0215 | 0.617 | 1.011 | 0.969, 1.054 |
| Cannabis vs. Controls | **0.047** | **0.1369** | **0.731** | **1.048** | **0.802, 1.371** |

Model 2

|  | Beta | S.E. | p | Odds Ratio | 95% CI OR |
| --- | --- | --- | --- | --- | --- |
| Magnitude | 0.218 | 0.0829 | 0.008 | 1.255 | 1.057, 1.463 |
| Probability | 0.279 | 0.0816 | 0.001 | 1.322 | 1.127, 1.551 |
| Expected Value | 0.276 | 0.1134 | 0.015 | 1.318 | 1.055, 1.646 |
| Trial Number | -0.015 | 0.0027 | <0.001 | 0.985 | 0.980, 0.990 |
| Gender | 0.126 | 0.0910 | 0.167 | 1.134 | 0.949, 1.356 |
| BDI | -0.006 | 0.0048 | 0.232 | 0.994 | 0.985, 1.004 |
| Av-Cigs/Day | -0.007 | 0.0069 | 0.294 | 0.993 | 0.979, 1.006 |
| Baseline button-pressing time | 0.011 | 0.0215 | 0.615 | 1.011 | 0.969, 1.054 |
| Cannabis vs. Controls | -0.005 | 0.1819 | 0.980 | 0.995 | 0.697, 1.422 |
| Cannabis vs Controls*Magnitude | **0.038** | **0.1053** | **0.715** | **1.039** | **0.845, 1.277** |

Model 3

|  | Beta | S.E. | p | Odds Ratio | 95% CI OR |
| --- | --- | --- | --- | --- | --- |
| Magnitude | 0.237 | 0.0853 | 0.006 | 1.267 | 1.072, 1.497 |
| Probability | 0.251 | 0.0904 | 0.005 | 1.285 | 1.077, 1.535 |
| Expected Value | 0.280 | 0.1136 | 0.014 | 1.323 | 1.059, 1.653 |
| Trial Number | -0.015 | 0.0028 | <0.001 | 0.980 | 0.980, 0.990 |
| Gender | 0.126 | 0.0910 | 0.1267 | 1.134 | 0.949, 1.356 |
| BDI | -0.006 | 0.0048 | 0.237 | 0.994 | 0.985, 1.004 |
| Av-Cigs/Day | -0.007 | 0.0069 | 0.294 | 0.993 | 0.979, 1.006 |
| Baseline button-pressing time | 0.010 | 0.0215 | 0.635 | 1.010 | 0.969, 1.054 |
| Cannabis vs. Controls | 0.004 | 0.1652 | 0.979 | 1.004 | 0.727, 1.389 |
| Cannabis vs. Controls*Probability | **0.054** | **0.1059** | **0.607** | **1.056** | **0.858, 1.300** |

Model 4

|  | Beta | S.E. | p | Odds Ratio | 95% CI OR |
| --- | --- | --- | --- | --- | --- |
| Magnitude | 0.237 | 0.0852 | 0.005 | 1.268 | 1.073, 1.498 |
| Probability | 0.278 | 0.0817 | 0.001 | 1.321 | 1.125, 1.550 |
| Expected Value | 0.215 | 0.1629 | 0.188 | 1.239 | 0.901, 1.705 |
| Trial Number | -0.015 | 0.0028 | <0.001 | 0.985 | 0.979, 1.006 |
| Gender | 0.128 | 0.0910 | 0.161 | 1.136 | 0.951, 1.358 |
| BDI | -0.006 | 0.0048 | 0.235 | 0.994 | 0.985, 1.004 |
| Av-Cigs/Day | -0.007 | 0.0070 | 0.289 | 0.993 | 0.979, 1.006 |
| Baseline button-pressing time | 0.010 | 0.0215 | 0.635 | 1.010 | 0.969, 1.054 |
| Cannabis vs. Controls | -0.025 | 0.1727 | 0.885 | 0.975 | 0.695, 1.368 |
| Cannabis vs. Controls*EV | **0.133** | **0.1769** | **0.451** | **1.142** | **0.808, 1.616** |
